# Remote Liver Ischemic Preconditioning Protects against Sudden Cardiac Death via an ERK/GSK-3β-Dependent Mechanism

**DOI:** 10.1371/journal.pone.0165123

**Published:** 2016-10-21

**Authors:** Zhaoyang Hu, Sheng Hu, Shuai Yang, Mou Chen, Ping Zhang, Jin Liu, Geoffrey W. Abbott

**Affiliations:** 1 Laboratory of Anesthesiology & Critical Care Medicine, Translational Neuroscience Center, West China Hospital, Sichuan University, Chengdu, Sichuan, China; 2 Department of Anesthesiology, Xijing Hospital, Fourth Military Medical University, Xi'an, Shanxi, China; 3 Bioelectricity Laboratory, Dept. of Pharmacology and Dept. of Physiology and Biophysics, School of Medicine, University of California Irvine, Irvine, California, United States of America; University of Minnesota, UNITED STATES

## Abstract

**Background:**

Preconditioning stimuli conducted in remote organs can protect the heart against subsequent ischemic injury, but effects on arrhythmogenesis and sudden cardiac death (SCD) are unclear. Here, we investigated the effect of remote liver ischemia preconditioning (RLIPC) on ischemia/reperfusion (I/R)-induced cardiac arrhythmia and sudden cardiac death (SCD) in vivo, and determined the potential role of ERK/GSK-3βsignaling.

**Methods/Results:**

Male Sprague Dawley rats were randomized to sham-operated, control, or RLIPC groups. RLIPC was induced by alternating four 5-minute cycles of liver ischemia with 5-minute intermittent reperfusions. To investigate I/R-induced arrhythmogenesis, hearts in each group were subsequently subjected to 5-minute left main coronary artery ligation followed by 20-minute reperfusion. RLIPC reduced post-I/R ventricular arrhythmias, and decreased the incidence of SCD >threefold. RLIPC increased phosphorylation of cardiac ERK1/2, and GSK-3β Ser9 but not Tyr216 post-I/R injury. Inhibition of either GSK-3β (with SB216763) or ERK1/2 (with U0126) abolished RLIPC-induced antiarrhythmic activity and GSK-3β Ser9 and ERK1/2 phosphorylation, leaving GSK-3β Tyr216 phosphorylation unchanged.

**Conclusions:**

RLIPC exerts a powerful antiarrhythmic effect and reduces predisposition to post-IR SCD. The underlying mechanism of RLIPC cardioprotection against I/R-induced early arrhythmogenesis may involve ERK1/2/GSK-3β Ser9-dependent pathways.

## Introduction

Sudden cardiac death (SCD) is a leading cause of mortality and morbidity worldwide, accounting for the loss of an estimated 325,000 adult lives each year in the United States alone. The majority of cases are the result of lethal arrhythmia arising from acute coronary ischemia[1]. Indeed, although beneficial, therapies such as thrombolytic agents, bypass surgery, or coronary balloon angioplasty, by restoring blood flow to the ischemic myocardium, may on the other hand provoke lethal arrhythmias including ventricular fibrillation within seconds after blood restoration. Therefore, identification of therapeutic approaches to enhance myocardial tolerance to ischemia/reperfusion (I/R) and reduce the incidence of ventricular tachycardia and SCD, is of great importance for patients with ischemic heart disease.

Ischemic preconditioning (IPC), a brief, sub-lethal ischemic insult directly to the heart, makes heart tissue relatively resistant to subsequent, more severe injury[2]. IPC-induced cardioprotection has been well characterized, with clear beneficial effects, including antiarrhythmic activity, observed in various animal models[3]. As the name suggests, “Remote ischemic preconditioning” (RIPC) involves transient interspersed cycles of ischemia–reperfusion stimulus applied in a remote limb or visceral organ (as opposed to the target organ itself). RIPC can protect target organs against subsequent sustained episodes of ischemia or I/R injury[4]. It is a promising strategy that induces incompletely understood endogenous protective mechanisms. Preclinical studies have been conducted to evaluate the potential role of RIPC on multi-organ salvage[5]. Beneficial tolerance can be achieved in the heart, with myocardial damage or infarct size minimized by inducing alternate cycles of ischemia-reperfusion preconditioning in arteries and vessels of the limbs, mesentery, intestine or kidney, as well as abdominal aorta in various animal models[6].RIPC may even protect the myocardium as effectively as direct cardiac IPC. Most, but not all, clinical studies[7] found attenuation in the release of cardiac enzymes reflecting myocardial injury in adults[8]or children[9] treated with transient limb I/R stimulus.

However, despite convincing evidence of its critical role in cardioprotection, the influence of RIPC on arrhythmogenesis during coronary artery disease progression or therapy *in vivo* remains incompletely understood. Although scattered reports indicated limb ischemic preconditioning raised the tolerance to reperfusion-induced arrhythmia[10], the question remains whether brief ischemic preconditioning of visceral organs such as the liver, the largest metabolic organ in the body, can reduce ventricular arrhythmogenesis and susceptibility to SCD. In addition, RIPC is a multifactorial process involving the interactions of multiple effectors and signaling mechanisms, and the molecular underpinnings of the protective effects are incompletely understood.

Several signaling pathways have been implicated in conventional modes of cardioprotection, including those of extracellular signal-regulated kinase (ERK) and glycogen synthase kinase-3β (GSK-3β). However, whether activation of ERK or GSK-3β is protective or detrimental for myocytes is controversial. GSK-3β is inactivated by phosphorylation at Ser9, but activated by phosphorylation at Tyr216. It is unknown if RLIPC-induced antiarrhythmic effects occur by modulating the phosphorylation status of Ser9 and/or Tyr216. Recent studies suggested that constitutive activation of GSK-3β may inhibit pathological hypertrophy[11]. However, others found that inactivation of GSK-3β, by phosphorylation at Ser9, induces cardioprotection against I/R injury[12] and that pharmacological inhibition of GSK-3β mimics the protective effects of IPC or RIPC[13].

In addition, little attention has been paid to signaling pathways that might disfavor post-I/R arrhythmogenesis. Given all these gaps in knowledge, our aims here were first to determine whether remote preconditioning of the liver (RLIPC), by cycles of I/R stimulus, protects the heart and reduces predisposition to SCD induced by subsequent severe coronary ischemia-reperfusion injury. Second, we aimed to elucidate whether RLIPC modulates GSK-3β at Ser9 and/or Tyr216, and further clarify the potential role of the ERK/GSK-3β pathway in antiarrhythmic activity afforded by remote ischemia preconditioning.

## Materials and Methods

### Animals

The study was approved by the Institutional Animal Care and Use Committee of Sichuan University (Sichuan, China) (Permit Number: 2015035A) and was carried out in accordance with the recommendations in the Guide for the Care and Use of Laboratory Animals of the National Institutes of Health (NIH Publication.8^th^ edition, 2011). Male Sprague-Dawley (SD) rats (10 weeks old; 220–250 g) were purchased from Chengdu Dashuo Experimental Animal Research Center (Chengdu, China). Animals were kept under a 12-h light–dark cycle at 20–25°C and a humidity of 60 ± 5% before experiments.

### Experimental protocol and surgical procedures

**Fig 1:**
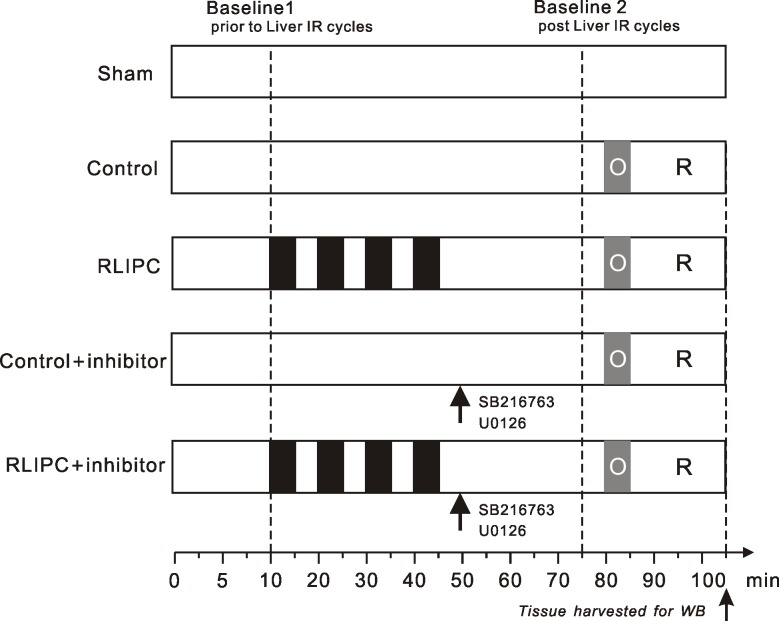
Experimental protocols. *A*ll groups were subjected to 5-min left main coronary artery occlusion followed by 20-min reperfusion except for the sham-operated group. Remote liver ischemia preconditioning (RLIPC) was induced by four cycles of 5 min of liver ischemia with 5 min intermittent reperfusions. Pharmacological inhibitors (SB216763 and U0126) were administered as a bolus 30 min prior to myocardial ischemia. Two baseline values were recorded: Baseline 1, ten minutes stabilization after instrumentation, and Baseline 2, twenty-five minutes post liver I/R stimulus.

Our experimental protocols are summarized in **Fig 1**. Details of the surgical implantation of instruments have been described previously[14–16]. Briefly, rats were anesthetized with intraperitoneal injection of sodium pentobarbital (50 mg/kg) and placed in a supine position. The adequacy of anesthesia was controlled by monitoring the loss of the corneal reflex and the lack of response to toe-pinching and was then continuously monitored through the evaluation of heart rate. After surgical preparation and instrumentation, rats were allowed 10 min stabilization before recording the first baseline values (Baseline 1). The anaesthetized rats were randomized to a sham-operated group (”Sham”, hepatic arterial and venous trunk were exposed without intervention, chests were opened without coronary artery ligation), control group (CON, no further hepatic intervention) or a remote liver ischemia preconditioning group (RLIPC). For the RLIPC rats, a median incision through the linea alba was made. After laparotomy, the portal vein, hepatic arterial and venous trunk were isolated and looped with a 3–0 silk[17]. Liver ischemic preconditioning was produced by four cycles of 5 min of liver ischemia with 5 min intermittent reperfusions (liver I/R cycles), i.e., clamping the vessel with an atraumatic microvascular clip to induce ischemia (ischemia period), and releasing the clip to initiate reperfusion (reperfusion period). Liver ischemia was confirmed by a change in the liver color. Following reperfusion, the liver color returned to pink.

To determine the influence of RLIPC on basic cardiac function, second baseline values (Baseline 2) were recorded 25 minutes after the accomplishment of all liver I/R cycles and before coronary ligation. Next, to assess the efficacy of RLIPC in protecting against cardiac ischemia/reperfusion (I/R)-induced ventricular arrhythmias and SCD, a segment of the proximal left main coronary artery was isolated and a 6–0 silk ligature (Ethicon, Somerville, NJ, USA) was placed around the vessel for production of coronary artery occlusion and reperfusion. Successful coronary artery occlusion was verified by the presence of regional dyskinesia and epicardial cyanosis in the ischemic zone. Reperfusion was verified by visual observation of an epicardial hyperemic response. Rats were subjected to 5 min left main coronary artery ligation followed by 20 min reperfusion, 5 min after Baseline 2 parameters were recorded. For the sham-operated animals, the left coronary artery was separated but not occluded. To delineate if the anti-arrhythmic effects mediated by RLIPC were dependent on GSK-3β/ERK signaling after reperfusion, 30 min prior to left main coronary artery ligation, GSK-3β inhibitor SB216763 (0.6 mg/kg) (Sigma, St. Louis, MO, USA) or ERK1/2 inhibitor U0126 (0.5 mg/kg) (Sigma, St. Louis, MO, USA) were applied to CON and RLIPC rats at the time of termination of the cycles of liver I/R preconditioning stimulus (CON+SB216763, CON+U0126, RLIPC+SB216763, RLIPC+U0126). Pharmacological inhibitors were intravenously bolus-injected into the femoral vein. Rat body temperature was maintained with a heating lamp.

At the end of each reperfusion period, the animals were euthanized with an overdose of sodium pentobarbital (200 mg/kg,i.p.)and death was monitored by cardiac activity and respiration. Blood samples were taken for blood serum biochemistry. In the sham group, the entire left ventricle was separated, whereas in other groups the coronary artery was re-occluded and 1% Evans blue (Sigma, St. Louis, MO, USA) was injected into the left ventricular cavity to delineate the ischemic area at risk (AAR) within the left ventricle. AAR was then collected and kept in -80°C freezer for western blotting analysis.

### Electrocardiography and hemodynamic analysis

After anesthesia, a standard limb lead II configuration electrocardiographic system was attached subcutaneously to the rats by needle electrodes. ST-segment (the period between the end of the QRS complex and the beginning of the T wave) duration was monitored throughout the experiment using a Powerlab/8sp system (AD Instruments, Colorado Springs, CO, USA). Changes inST segment duration were quantified offline usingLabChart7.2.1 software (AD Instruments, Colorado Springs, CO, USA).

Hemodynamics were recorded from the time period of Baseline 1 to Baseline 2 and before or after myocardial ischemia. The fur in neck regions of the rats was then removed and disinfected with ethanol (70%). The skin were cut and the subcutaneous tissues were freed to expose the right carotid artery next to the trachea. A 20-G heparin-filled catheter (Spacelabs Medical, Inc., Redmond, WA, USA) was introduced into the right carotid artery and advanced into the left ventricle for measurement. Hemodynamic parameters including left ventricular systolic pressure (LVSP), left ventricular end diastolic pressure (LVEDP), and maximum rate of increase/decrease in left ventricular pressure (±dP/dtmax) were recorded by the connected calibrated pressure transducer with physiologic recorder (Taimeng, Chengdu, China). Data were analyzed offline with Biolap 420F software (Taimeng, Chengdu, China).

### Arrhythmia analysis

The following arrhythmia events were recorded during the entire 20 minutes of reperfusion injury period after coronary artery ischemia and quantitated offline by LabChart7.2.1 software (AD Instruments, Colorado Springs, CO, USA): (1) Number of rats in sinus rhythm (without any arrhythmia); (2) Number of rats with ventricular tachycardia (VT); (3) Number of rats with sustained VT (>1min VT) (SVT); (4) Number of rats with polymorphic VT (PVT); (5) Number of rats with ventricular fibrillation (VF); (6) Number of rats with sudden cardiac death (SCD), in which there was a complete heart block during these 20minutes reperfusion period; (7) Number of rats with AV block (AVB); (8) Starting time of the first run of VT or VF; (9) Duration of VT; (10) the longest episode of VT duration (LVT); (11) Incidence of VF.

### Blood serum biochemistry

After 20 min of reperfusion, blood samples were taken from the heart, transferred to precooled tubes, and centrifuged at 1,000 *g* for 10 min at 4°C immediately after collection. The serum were frozen at -20°C until assay. Serum potassium concentration was measured automatically using an ABL800 Flex analyzer (Radiometer Medical ApS, Brønshøj, Denmark).

### Western blotting

Hearts were taken after 20 min of reperfusion and were homogenized with a pestle grinder system (Fisher Scientific, Hampton, NH, USA) in RIPA buffer containing 50 mMTris-HCl (pH7.4), 150 mMNaCl, 1% NP-40, 1 mM EDTA, 0.25% sodium deoxycholate, phosphatase inhibitor cocktail (Sigma-Aldrich, St. Louis, MO, USA), and a protease inhibitor cocktail (Sigma-Aldrich, St. Louis, MO, USA). Then, homogenates were centrifuged for 10 min at 10,000 *g*. Debris was removed and the supernatant was retained. BCA method was used for protein concentration determination (Pierce, Rockford, IL, USA). Equal amounts of protein (15 μg protein/lane) were heated to 80°C and were resolved on a 12% SDS-PAGE gel and transferred onto nitrocellulose membranes (VWR, Batavia, IL, USA). The membrane was blocked at room temperature and then was incubated with primary antibodies raised against phosphorylated extracellular signal-regulated kinase1/2 (ERK1/2) (Thr202/Tyr204), total ERK1/2 (rabbit, 1:1000, Cell Signaling, Danvers, MA, USA), phosphorylated glycogen synthase kinase-3β(Ser9) (p-GSK-3β Ser9, rabbit, 1:1000, Cell Signaling) and phosphorylated glycogen synthase kinase-3β(Tyr216) (p-GSK-3β Tyr216, rabbit, 1:500, Santa Cruz Biotechnology, Santa Cruz, CA, USA), total GSK-3β (rabbit, 1:1000, Cell Signaling), followed by incubation with horseradish peroxidase (HRP)-conjugated goat anti-rabbit IgG secondary antibody (Bio-Rad, Hercules, CA, USA). Immunoreactive bands were visualized by chemiluminescence ECL (Millipore, Billerica, MA, USA). Signals were obtained by AmershamImager 600 system (GE healthcare, Little Chalfont, UK). Band densities were measured using ImageJ Data Acquisition Software (National Institutes of Health, Bethesda, MD, USA). Phosphorylation-signal densities were normalized to the corresponding total protein-signal densities. Results were normalized to those of control hearts.

### Statistical analysis

All values are given as mean ± SEM. The Kolmogorov-Smirnov test was used to verify the assumption of normal distribution. Comparisons between two values were performed using unpaired two-tailed student’s t-tests. Fisher’s exact test was employed to compare numbers of rats falling into one of two groups. One-way ANOVA was employed for multiple comparisons among different groups. Homogeneity of variance was tested by Levene’s test. If variances were equal, the Newman-Keuls test was examined post hoc for multiple comparisons; otherwise, Dunnett’s T3 test was applied. Two-way repeated-measures analysis of variance (ANOVA) was applied to identify the statistical difference of hemodynamic changes or ST heights over time, with group as a between-subjects factor and time as a within-subjects factor. Group × time interactions were tested. Sphericity assumption was determined by Mauchly’s test. If not applicable, a Greenhouse–Geisser correction was applied for degrees-of-freedom adjustment. When differences were detected for group-time interactions, the Bonferroni correction procedure was used for multiple comparisons at individual time points between groups; otherwise, global conclusions were made and comparisons between times were conducted. All P-values were two-sided. *P*< 0.05 was considered statistically significant.

## Results

### RLIPC elevates ST prior to coronary ischemia

**Fig 2:**
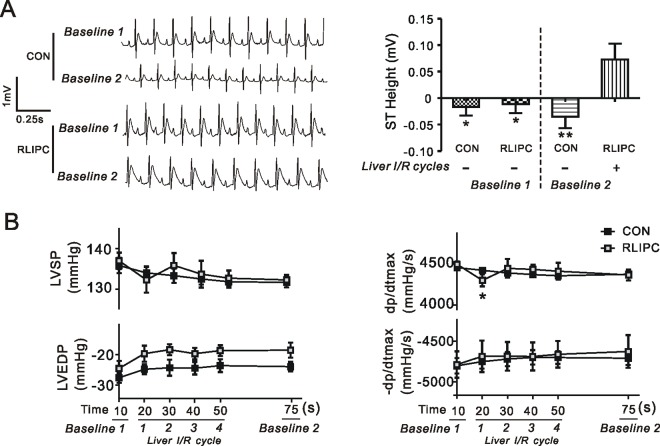
RLIPC elevates ST segment prior to induction of cardiac ischemia in rat model of arrhythmia. ***A)* Left**, typical ECGs from CON and RLIPC rats during baseline periods showing elevated ST segment in RLIPC-treated rats. **Right**, quantification of parameters from ECGs showing elevated ST segment in RLIPC rats post liver I/R stimulus (*n =* 9–10, each group). *P<0.05, **P<0.01, *vs*. RLIPC in the presence of liver I/R cycles. ***B*)** Variables recorded during the experimental protocol investigating the effect of liver I/R stimulus on hemodynamics prior to coronary artery ligation. LVSP, left ventricular systolic pressure; LVEDP, left ventricular end-diastolic pressure; ±dP/dtmax, maximum rate of increase/decrease in left ventricular pressure. Data are the mean±SEM (*n = 6–7* in each group). *P<0.05 compared with values at Baseline 1. Baseline 1 indicates the first baseline value after 10 minutes stabilization; Baseline 2 indicates the second baseline value obtained 5 minutes prior to coronary ligation. Hearts in RLIPC group experienced four cycles of I/R stimulus, indicated as 1, 2, 3, 4.

ECG analysis revealed striking ST elevation in rats after 4 cycles of liver artery I/R (P = 0.0055, **Fig 2A**). Systemic hemodynamic parameters were not altered between two groups under baseline conditions, nor during RLIPC apart from depression of the systolic function in dP/dtmax (P = 0.033 versus baseline) during the first liver I/R cycle (**Fig 2B**).

### RLIPC reduces susceptibility to post-ischemic ventricular arrhythmias

**Fig 3:**
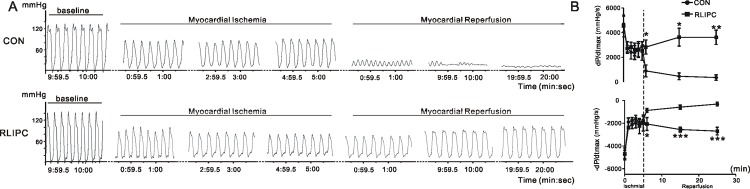
RLIPC improves cardiac function post-ischemic arrhythmia. **A)** Representative hemodynamic traces from CON and RLIPC rats prior to- or post-myocardial ischemia showing preservation of cardiac function with liver ischemic preconditioning. **B)** Left ventricular ±dP/dtmax values measured during and after myocardial ischemia in control and RLIPC-treated rats; *n* = 6–8 per group. *P<0.*05* and ***P<0.*001* vs. CON.

To explore the effect of RLIPC on cardiac susceptibility to post-ischemic ventricular arrhythmias, rats in either group were exposed to a 5-minute left main coronary ligation followed by 20-minute reperfusion. Coronary artery ligation was accompanied by a similar reduction of systolic function in dP/dtmax, and an increase in -dP/dtmax in control and RLIPC-treated rats. However, significant differences in ±dP/dtmax were observed between RLIPC-treated and non-treated hearts after reperfusion injury, indicating preservation of cardiac function with liver ischemic preconditioning (**Fig 3A and 3B**). All control rats (16 out of 16) exhibited one or more arrhythmias including ventricular tachycardia (VT), polymorphic ventricular tachycardia (PVT), or atrioventricular block (AVB), throughout reperfusion. In contrast, 5 out of 14 RLIPC rats remained in sinus rhythm (no arrhythmia) during reperfusion, while the remainder exhibited arrhythmia (P = 0.014). Although VT was prevalent in all control rats (16/16), only 64% of RLIPC rats (9/14) developed VT (P = 0.014), with a decreased incidence of sustained VT (>1 minute) within 20 minutes from 87.5% (CON) to 21.4% (P = 0.0006). Moreover, 15 of 16 control rats exhibiting monomorphic VT gradually degenerated into PVT, which is strongly linked to fatal ventricular fibrillation (VF) or SCD in patients[18]. The incidence of PVT was much lower in RLIPC rats (4/14, or 28.6%; P = 0.0004). Only 1 rats in the control group showed solely monomorphic VT and eventually returned to sinus rhythm (1/16), whereas the majority of rats in the RLIPC group with arrhythmia (11/14) presented solely with monomorphic VT and returned to sinus rhythm within 20 minutes (P = 0.0001 by Fisher exact test).

**Fig 4:**
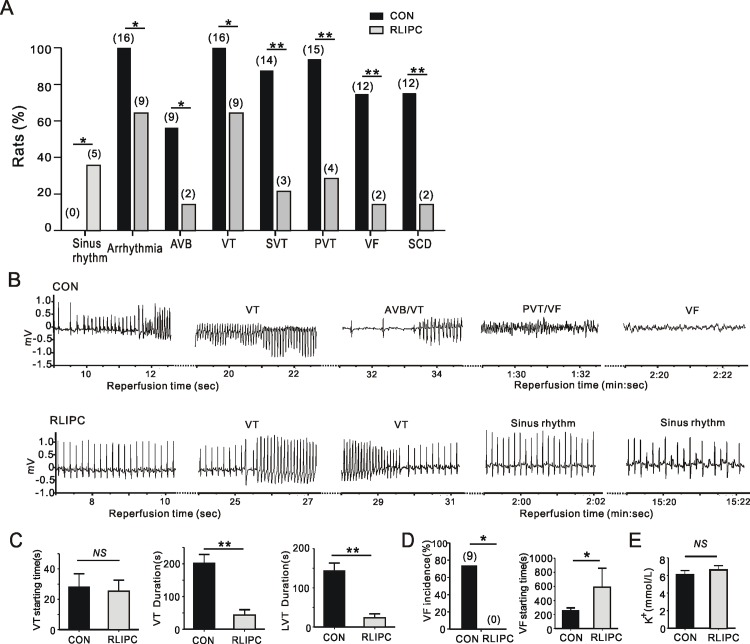
RLIPC confers cardioprotection against post-ischemic arrhythmia and SCD. **A)** Quantification of the incidence of different cardiac arrhythmia characteristics and mortality during post ischemia reperfusion in CON (*n* = 16) and RLIPC (*n* = 14) rats. Numbers of animals per category are indicated in parentheses. Sinus rhythm, remained in sinus rhythm; AVB, AV block; VT, ventricular tachycardia; SVT, sustained VT (>1min VT); PVT, polymorphic VT; VF, ventricular fibrillation; SCD: sudden cardiac death. *P<0.*05* and **P<0.*01* vs. CON. **B)** Representative surface ECGs from CON and RLIPC rats during reperfusion showing greater severity of arrhythmia in the former. **C)** Mean VT parameters including the onset time of first VT episode after reperfusion (left) and durations of VT (middle) and the longest episode of VT duration (LVT) (right) for control (*n* = 16) and RLIPC (*n* = 14) rats. Rats without VT were indicated as 0 duration. **P<0.*01 vs CON*, *NS*: *P>0*.*05* between two groups. **D)** Left, VF incidence during the first 5 minutes of reperfusion for CON (n = 16) and RLIPC (*n* = 14) rats. Numbers in parentheses indicate the numbers of rats exhibiting VF; right, the starting time of the first run of VF after the onset of reperfusion in CON (*n* = 16) and RLIPC (*n* = 14) rats. *P<0.*05* vs. CON. **E)** Mean serum K^+^ concentration for CON and RLIPC rats after 20min reperfusion; *n* = 8 per group. *NS*: *P>*0.05 between two groups.

Importantly, there was a striking protective effect of RLIPC against SCD (2 death/14 RLIPC rats, compared to 15 death/16 control rats; P = 0.0001). In both groups, death was caused by an arrhythmic cardiac arrest due to VF or severe AVB.The fraction of rats exhibiting VF was greater in the control group (12/16) than in the RLIPC group (2/14) (P = 0.0013), and AV block also occurred more frequently in control rats (9/16) than in RLIPC rats (2/14; P = 0.0259) *(***Fig 4A***)*. Representative ECG tracings from control and RLIPC ratsillustrate the lower predisposition to reperfusion VT/VF of RLIPC rats compared to control rats *(***Fig 4B***)*.

**Fig 5:**
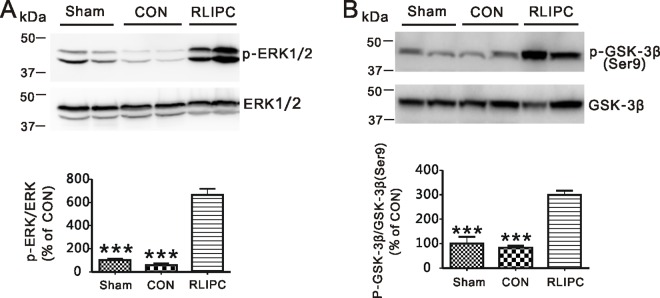
RLIPC phosphorylates ERK1/2 and GSK-3β post cardiac IR injury. **A)** Left, Western blots of ventricular phosphorylated ERK1/2 and total ERK1/2 isolated from sham, control and RLIPC rats after 20 minutes of reperfusion; right, band density of p-ERK/ERK (*n* = 6–8 per group). ***P<0.*001* vs. RIPC. **B)** Left, Western blots of ventricular phosphorylated GSK-3β(Ser9) and total GSK-3β isolated from sham, control and RLIPC rats after 20 minutes of reperfusion; right, band density of p-GSK-3β(Ser9)/GSK-3β (*n* = 6–8 per group). ***P<0.*001* vs. RIPC.

During the post-ischemic reperfusion period, rats in the control group showed increased propensity for, and duration of, ventricular arrhythmogenesis. Although the starting time of the first recorded VT episode was similar in either group during the reperfusion period (P = 0.8415), mean VT duration was >3-fold longer in control rats (201.8 ± 26.6 s) compared to RLIPC rats (67.1 ± 22.4 s, P = 0.0023). Furthermore, control rats showed markedly prolonged continuous episodes of long episode VT (mean duration 143.1 ± 20.0 s), the longest episode lasting 280 s. In contrast, RLIPC successfully reduced the mean longest episode of VT duration to 23.4 ± 10.5 s (P = 0.0001) (**Fig 4C**). Notably, a total of 12 control rats developed VF in this experiment (12/16), nine of which started to exhibit VF within the first 5 minutes after the onset of reperfusion, while none of the two RLIPC rats that ultimately developed VF exhibit VF in that early phase of reperfusion. In addition, RLIPC delayed the onset of the first run of VF after commencing reperfusion, from 251.9 ± 43.4 s in the control group to 587.5 ± 272.5 s (P = 0.033) (**Fig 4D**). We did not observe alteration of serum potassium concentration after reperfusion; thus, the preconditioning effect was unlikely to arise from changes in K^+^ homeostasis (**Fig 4E**). The ERK and GSK-3β-related cell survival signaling pathway is known to be activated by preconditioning stimulus[13, 19]. We did not detect a difference in total ERK1/2 and GSK-3β protein levels between control and RLIPC ischemic ventricles isolated after 20 minutes of reperfusion. In contrast, prior RLIPC dramatically increased phosphorylation of ventricular ERK1/2 (Thr202/Tyr204) (P<0.0001) and GSK-3β (Ser9) (P<0.0001) compared to non-RLIPC (control) hearts following cardiac reperfusion. No differences were detected between sham-operated and control rats (**Fig 5A and 5B**).

### Inhibition of ERK and GSK-3β impairs the anti-arrhythmic action of RLIPC

**Fig 6:**
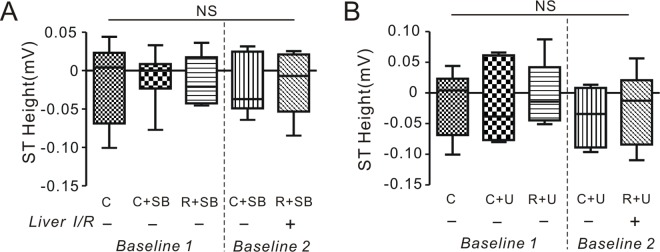
The influence of pharmacological inhibitors on ST elevation in rats. **A)** The change of ST height in the absence (-) or presence (+) of liver I/R preconditioning stimulus when applying GSK-3β inhibitor SB216763 (*n* = 6–9 per group). *NS*: *P>0*.*05* among groups. ST height for control group is repeated from Fig 2 for comparison. C, control; R, RLIPC; SB, SB216763. **B)** The change of ST height in the absence (-) or presence (+) of liver I/R preconditioning stimulus when applying ERK inhibitor U0126 (*n* = 6–9 per group). *NS*: *P>0*.*05* among groups. ST height for control group is repeated from Fig 2 for comparison. C, control; R, RLIPC; U, U0126.

To further explore the respective roles of ERK and GSK-3β in RLIPC-induced cardioprotection, we applied pharmacological inhibitors after the liver I/R stimulus but prior to cardiac I/R. As shown in **Fig 6**, in contrast to data obtained in the absence of inhibitors (**Fig 2**), however, SB216763 or U0126 pretreatment ameliorated the ST segment elevation we had previously observed for RLIPC rats (**Fig 6A and 6B; compare to Fig 2**). Thus, in this system, disruption of ERK1/2 or GSK-303B2 signaling ahead of a more severe myocardial ischemia episode reversed the ST segment elevation caused by RLIPC preconditioning.

**Fig 7:**
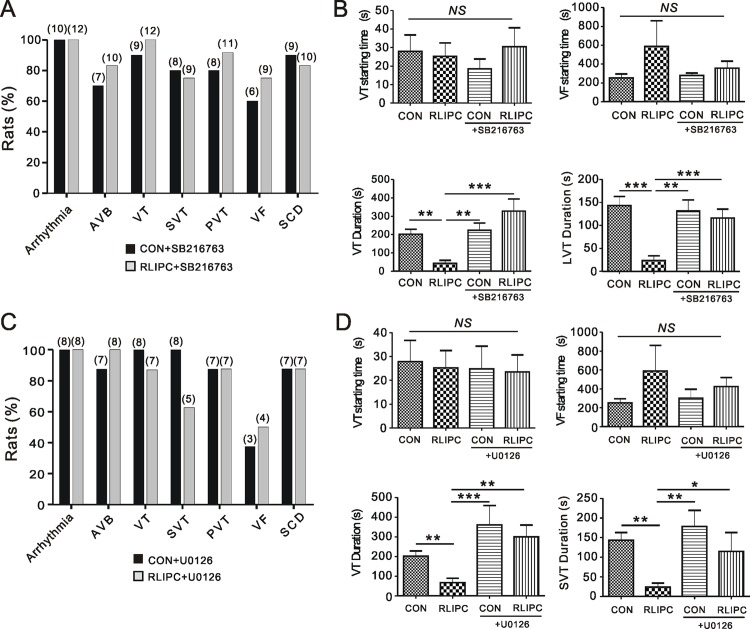
Pharmacological inhibition of ERK or GSK-3β abolishes RLIPC-induced anti-arrhythmic cardioprotection. **A)** The effect of inhibiting GSK-3β on post-reperfusion arrhythmia characteristics and mortality in CON (*n* = 10) and RLIPC (*n* = 12) rats upon administration of GSK-3β inhibitor SB216763 prior to coronary ligation. Numbers of animals per category are indicated in parentheses. CON, control; RLIPC, remote liver ischemia preconditioning. AVB, AV block; VT, ventricular tachycardia; SVT, sustained VT (>1min VT); PVT, polymorphic VT; VF, ventricular fibrillation; SCD: sudden cardiac death. **B)** Mean values for onset time of first VT (upper left)/VF (upper right) episode after reperfusion and durations of VT (lower left), the longest episode of VT duration (LVT, lower right) for control (*n* = 10) and RLIPC (*n* = 12) rats upon GSK-3β inhibitor SB216763 application. Values for control and RLIPC rats are repeated from Fig 3 for comparison. *NS*, no significant difference, **P<0.*01*, ***P<0.*001* compared withRLIPC (by One-way ANOVA). **C)** The effect of administering ERK inhibitor U0126 to CON (*n* = 8) and RLIPC (*n* = 8) rats on post-reperfusion arrhythmia characteristics and mortality. Values in parentheses indicate the numbers of rats per category. **D)** Mean values for onset time of first VT (upper left)/VF (upper right) episode after reperfusion and durations of VT (lower left), the longest episode of VT duration (LVT, lower right) for control (*n* = 8) and RLIPC (*n* = 8) rats upon ERK inhibitor U0126 application. Values for control and RLIPC rats are repeated from Fig 3 for comparison. *NS*, no significant difference, *P<0.*05*, **P<0.*01*, ***P<0.*001* compared withRLIPC (by One-way ANOVA).

Next, we quantified the effects on post cardiac I/R arrhythmogenesis of pretreatment with either inhibitor in control and RLIPC rats. Strikingly, SB216763 and U0126 each robustly disrupted RLIPC-induced post-ischemic cardioprotection, such that all inhibitor-treated RLIPC and control rats exhibited arrhythmias after reperfusion. While non-pretreated RLIPC rats were less susceptible to post-I/R arrhythmia compared to control rats, SB216763 pretreatment eliminated this difference, resulting in similar incidence, between CON and RLIPC groups, of SCD (P = 1.00), VT (P = 0.46), SVT (P = 1.00), PVT (P = 0.57), VF (P = 0.65) and AVB (P = 0.62) (**Fig 7A**). Inhibition of GSK-3β did not change the latency to the first episode of VT, but it eliminated the delay in onset of the first episode of VF that was achieved by RLIPC without SB216763 pretreatment (P>0.05, **Fig 7B; compare with Fig 4**). SB216763 pretreatment also increased post-I/R VT duration and the longest episode of VT duration compared to rats exposed to RLIPC preconditioning alone (*P<0*.*01*) (**Fig 7B**). Likewise, ERK1/2 inhibitor U0126 nullified the cardioprotective effects of RLIPC. There was no difference between treated control and RLIPC rats with respect to the incidence of SCD (*P = 1*.*00*), VT (*P = 1*.*00*), SVT (*P = 0*.*20*), PVT (*P = 1*.*00*) or AVB (*P = 1*.*00*) (**Fig 7C**), or the latency to VT or VF (*P>0*.*05*) (**Fig 7D**). In addition, U0126 increased the duration of VT (*P<0*.*01*) or the longest VT episode (*P<0*.*05*) in RLIPC rats (**Fig 7D**).

### The anti-arrhythmic effects of RLIPC at reperfusion are mediated by ERK and GSK-3β dependent mechanism

**Fig 8:**
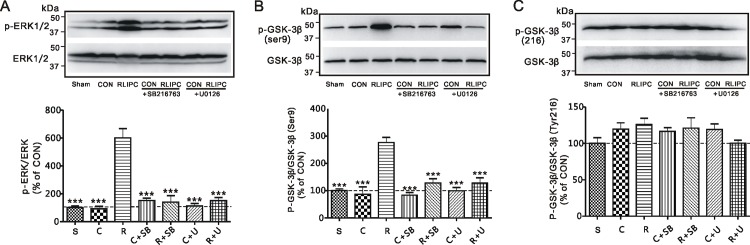
The influence of pharmacological inhibitors on cardiac ERK and GSK-3β phosphorylation status in rats. **A) Upper panel,** Representative western blots of phosphorylated ERK1/2 and ERK1/2 isolated from CON and RLIPC rats in the presence or absence of pharmacological inhibitors. CON, control; RLIPC, remote liver ischemia preconditioning. **Lower panel**, Quantification of p-ERK/ERK protein band density; *n* = 6–12, each group. S, sham-operated group, C, control; R, RLIPC; SB, SB216763; U, U0126. ***P<0.*0001* compared with RLIPC (by One-way ANOVA). All other group comparisons showed *P>0*.*05*. **B) Upper panel,** Representative western blots of phosphorylated GSK-3β(Ser9) and total GSK-3β isolated from control and RLIPC rats in the presence or absence of pharmacological inhibitors. **Lower panel**, Quantification of p-GSK-3β(Ser9)/GSK-3β protein band density; *n* = 5–10, each group. S, sham-operated group, C, control; R, RLIPC; SB, SB216763; U, U0126. ***P<0.*0001* compared with RLIPC (by One-way ANOVA). All other group comparisons showed P>0.05. **C) Upper panel,** Representative western blots of phosphorylated GSK-3β(Tyr216) and total GSK-3β isolated from control and RLIPC rats in the presence or absence of pharmacological inhibitors. **Lower panel**, Quantification of p-GSK-3β(Tyr216)/GSK- 3β protein band density; *n* = 6–9, each group. S, sham-operated group, C, control; R, RLIPC; SB, SB216763; U, U0126. All group comparisons showed *P>0*.*05*.

To investigate the possibility of functional interaction between ERK and GSK-3β, we compared the effects of their antagonism (prior to myocardial ischemia insult) on each other’s phosphorylation. There was no difference among control groups in the presence or absence of inhibitors (*P>0*.*05*, **Fig 8**). ERK and GSK-3βwere similarly phosphorylated in control and sham-operated hearts. However, RLIPC-induced phosphorylation of ERK was completely blocked by applying U0126, an inhibitor of ERK (*P<0*.*0001*, **Fig 8A**). Strikingly, administration of SB216763, a GSK-3β inhibitor, also caused a significant decrease (4.3-fold) in ventricular ERK phosphorylation compared to rats exposed to RLIPC alone, diminishing it to a level similar to that of hearts in the control group (*P<0*.*0001*, **Fig 8A**).

Additionally, although GSK-3βphosphorylation level was unaltered in control hearts when compared to sham hearts(*P>0*.*05*), direct inhibition of GSK-3β with SB216763 eliminated the 2.8-fold greater GSK-3β (Ser9) phosphorylation we had previously observed with RLIPC (RLIPC+SB216763 *vs*. RLIPC, *P<0*.*0001*,**Fig 8B**). Importantly, phosphorylation of GSK-3β (Ser9) was also impaired by ERK inhibitor U0126, which halved the fractional GSK-3β (Ser9) phosphorylation level from 1.16± 0.07 (RLIPC) to 0.54 ± 0.08 arbitrary units (RLIPC+U0126) (*P<0*.*0001*, **Fig 8B**). In contrast, ventricular GSK-3β (Tyr216) phosphorylation was unaffected by RLIPC, SB216783 or U0126 (**Fig 8C**). Thus, RLIPC-induced cardiac ERK and GSK-3β activities were interdependent, while GSK-3β Ser9 phosphorylation occurred independently of Tyr216 phosphorylation in RLIPC-induced anti-arrhythmic activity.

## Discussion

In the present study, we demonstrate that RLIPC effectively reduces myocardial vulnerability to post-I/R arrhythmias and SCD via ERK/GSK-3β signaling mechanism.

Remote ischemic preconditioning (RIPC), a transient period of blood flow interruption followed by reperfusion in remote organ or limbs, has been shown to protect hearts by release of biochemical messengers into the circulation or by activation of various pathways[4, 5]. Abundant studies have been carried out to show the benefit to the heart of prior brief ischemia of the mesentery, limb and kidney[6]. However, despite the well-known role of RIPC in limiting infarct size against I/R injury[20], the influence of RIPC on ameliorating post-I/R arrhythmogenesis is much less well understood. In addition, compared to other organs, preconditioning stimulus produced in the liver may introduce additional features such as reducing inflammation and promoting hepatic regeneration[21]. Prior studies provided evidence that remote protection can be achieved *in vivo* in lung[22] and kidney[23] by brief liver ischemia, and *ex vivo* in the heart[24]. Our current results confirm and expand upon previous findings involving limb RIPC[10]^,^[25], add novel mechanistic information with discovery of involvement of ERK/GSK-3β signaling in cardioprotection, and suggest unique mechanistic features of RLIPC anti-arrhythmogenesis early in reperfusion.

The ST vector magnitude was shown to increase during preconditioning cycles of local cardiac ischemia-reperfusion stimuli [26]. Interestingly, we found that ST level was elevated after RLIPC before any cardiac manipulation. It is possible that the heart actually received classic ischemic preconditioning via RLIPC, thus became tolerant to the following severe ischemic insult. The basic mechanisms responsible for the ST elevation during liver ischemic preconditioning in the present study is unclear. Data from the studies using channel inhibitors suggest that K-ATP channels may contribute to ST elevation within the heart [27]. This electrical inhomogeneity could reflect the potential underlying cardioprotective mechanism of RLIPC. Therefore, future studies are promoted to elucidate the actual contributions of ST elevation to the RLIPC-induced antiarrhythmic effects observed in the current study. Meanwhile, since we did not detect any hyperkalemia in RLIPC-treated rats after reperfusion, the significance of RLIPC-induced cardioprotection in the present study is less likely due to proarrhythmic activity exerted by elevated potassium concentration[28].

Although progress has been made in trying to elucidate the cardiac signaling components contributing to the infarct-limiting effects of RIPC, little is known about the mechanisms underlying RIPC-induced antiarrhythmic effects. Phosphorylation of GSK-3β at Ser9 or Tyr216 results in the inhibition or enhancement of GSK-3β activity, respectively[29]. Inhibition of GSK-3β by Ser9 phosphorylation has been shown to suppress mPTP opening and enhance cell survival by inducing infarct size limitation against I/R injury in local[30, 31] or remote ischemic preconditioned hearts[32]^,^[33]. Our phosphorylation data are consistent with these previous studies, as we found that in a model of severe arrhythmia, pretreatment with liver I/R stimulus enhanced GSK-3β Ser9 phosphorylation, which decreased the incidence of SCD and diminished the predisposition to arrhythmias after reperfusion. We also found no effect of RLIPC alone on GSK-3β Tyr216 phosphorylation post-I/R, concordant with previous studies concerning opioid-induced cardioprotection during reperfusion[34]. This would suggest that similar to opioids[34] or insulin[35], RLIPC-induced cardioprotection was exerted via selectively modulating GSK-3β at Ser9, but not Tyr216. However, we found SB216763, a GSK-3β inhibitor, nullified the anti-arrhythmic benefits of RLIPC in our rat arrhythmia model, and suppressed RLIPC-induced GSK-3β Ser9 phosphorylation, without affecting GSK-3β Tyr216 phosphorylation. The finding is in contrast to work by others which showed pharmacological inhibition of GSK activity limited infarct size following I/R[19]. This detrimental effect is in some ways reminiscent of prior studies that showed inhibition of GSK-3β exacerbated myocardial damage[11, 36]^,^ [37]. Interestingly, others concluded that GSK3 inactivation is not required for ischemic preconditioning, in a mouse infarct model[38].In addition, the mechanism underlying RLIPC may differ from that of limb preconditioning, which shows no involvement of the RISK pathway in the antiarrhythmic process[10].

Extracellular signal-regulated kinases (ERK1/2), one of the four major kinase cascades of mitogen-activated protein kinases (MAPKs), are involved in a diverse repertoire of biological events. Studies have implicated that various stimuli including ischemic conditioning could phosphorylate ERK1/2, which activates ERK1/2, leading to infarct reduction[33, 39]. Our present study confirms a more favorable role for the ERK pathway in RLIPC-induced antiarrhythmic cardioprotection, and is in agreement with these previous studies by showing that activation of ERK1/2 by RLIPC leads to cardioprotection against I/R injury. However, the cardioprotective effects of ERK inhibition are complex. It has been reported that increased apoptosis following ERK inhibition was detected either in cultured neonatal rat ventricular myocytesor in the isolated perfused heart *ex vivo*[40]. Thus, disruption ofERK1/2activity was considered to be a detrimental factor to some extent. Furthermore, some prior studies showed that pharmacological inhibitors can disturb cardioprotective effects offered by IPC[41, 42] or RIPC[6, 33]. Similarly, here, we found that inhibition of ERK by U0126 impaired the anti-arrhythmic effect offered by RLIPC.

ERK1/2 and GSK-3β have been identified as major mediators of cardioprotection regardless of whether their activation could be detected in the heart after remote ischemic preconditioning[5, 19]. The combination of effects we observed using the Arrhythmia model, i.e., abrogation by ERK1/2 and GSK-3β inhibitors both of RLIPC-induced anti-arrhythmic cardioprotection, and of ERK1/2 and GSK-3β Ser9 phosphorylation, directly implicates a pathway involving these two proteins in anti-arrhythmic effects of RLIPC. Reports showed that activated ERK1/2 leads to phosphorylation of GSK-3β, which inactivates GSK-3β. U0126inhibits ERK activity and therefore causes dephosphorylation of GSK-3β[19]. It is not surprising that we found U0126 attenuated phosphorylation of GSK-3β, since ERK is recognized as a kinase upstream of GSK-3β. Unexpectedly, we found that ERK phosphorylation was also inhibited by SB216763, which may add to the controversial debate concerning the interaction of ERK- GSK-3β signaling cascade in liver conditioning-induced anti-arrhythmic protection. Furthermore, evidence indicates direct association between GSK-3β and ERK1/2 molecules[43], and treatment with GSK-3β inhibitors could increase ERK1/2 phosphorylation, thereby triggering ERK1/2 activation in non-cardiac cell lines[44, 45]. Thus, GSK-3β was proposed as a negative regulator of ERK1/2. Our findings may also suggest non-canonical action of SB216763 and/or GSK-3β itself within the context of the Arrhythmia protocol, because GSK-3β Ser9 phosphorylation is inhibitory, yet SB216763 prevented the otherwise robust GSK-3β Ser9 phosphorylation we observed after RLIPC. This unexpected effect is one potential cause of the abrogation by SB216763 of the RLIPC anti-arrhythmic action. While some prior studies also showed that pharmacological inhibitors can disturb cardioprotective effects offered by IPC[41, 42] or RIPC[6, 33], the authors are not aware that disruption ofSer9 phosphorylation by SB216763was a factor, nor are we aware of any other studies demonstrating protocol-dependent capacity of SB216763 to prevent GSK-3β Ser 9 phosphorylation. After all, GSK-3β is a multifunctional protein that can act differently depending on contexts including cellular location and toxic insult, the balance between GSK-3β Ser9 and Tyr216 phosphorylation, and even depending on phosphorylation state in a manner independent of activation state[46].

We acknowledge potential limitations of this study. First, other signaling pathways, such as the JAK/STAT pathway, also participate in the cardioprotective cascade. Signal transducer and activator of transcription-3 (STAT-3), part of the survivor activating factor enhancement (SAFE) pathway, has been shown to be activated upon either local [47] or remote [33] ischemic stimuli. Pharmacologic inhibition of STAT-3 was previously found to abolish the cardioprotective effect exerted by RIPC [33]. The possibility was not experimentally addressed here, but future studies could address whether the STAT pathway is involved in liver ischemic preconditioning-induced anti-arrhythmogenesis activity, and if there is any crosstalk in this context with the ERK/GSK pathway. Second, we tested only one RLIPC protocol using four cycles of 5 min ischemia/5 min reperfusion prior to coronary ligation and one specific animal model, thus, it remains unknown whether other RLIPC strategies can affect arrhythmogenesis or whether RLIPC is cardiac protective in other cardiac disease models. Third, SB216763 and U0126 were employed as GSK and ERK inhibitors to identify the contribution of these two proteins and their potential functional interactions. While the two inhibitors are widely used and accepted to be specific to the proposed target molecules, it remains possible that they could impact additional signaling molecules that might also modulate the interaction of ERK1/2 with GSK-3β, or other pathways. Fourth, liver-derived endocrine (humoral) factors, neural influences or inflammatory factors may possibly contribute to RLIPC-induced pathways and lead to or influence ERK1/2 and GSK-3β phosphorylation, and could be the topic of a future study. Finally, despite the established role of volatile anesthetics in inducing ischemia preconditioning (APC) on their own[16, 48], anesthetic regimes have controversial effects on RIPC depending on the background anesthetics chosen in various surgery strategies and clinical settings[49]. To avoid potential involvement of APC in this study, we alternatively used intraperitoneal injection of sodium pentobarbital for anesthesia, which is not known to exert cardioprotective effects. Accordingly, using similar anesthetic protocols between groups in the present study, we showed that RLIPC successfully delivered antiarrhythmic cardioprotection whereas control groups were severely affected by arrhythmias.

## Conclusions

Pretreatment with liver ischemic preconditioning renders the heart less susceptible to subsequent severe myocardial ischemia and reperfusion-induced ventricular arrhythmia, which may occur as a consequence of phosphorylation of critical myocardial kinases ERK1/2 and GSK-3β. However, pharmacologic inhibition of either of these kinases abolished the RLIPC-induced anti-arrhythmic effects. The underlying mechanism of RLIPC induced cardioprotection may involve ERK1/2/GSK-3β Ser9-dependent pathways.
